# Multistate Outbreak of SARS-CoV-2 Infections, Including Vaccine Breakthrough Infections, Associated with Large Public Gatherings, United States

**DOI:** 10.3201/eid2801.212220

**Published:** 2022-01

**Authors:** Radhika Gharpure, Samira Sami, Johanna Vostok, Hillary Johnson, Noemi Hall, Anne Foreman, Rebecca T. Sabo, Petra L. Schubert, Hanna Shephard, Vance R. Brown, Ben Brumfield, Jessica N. Ricaldi, Andrew B. Conley, Lindsay Zielinski, Lenka Malec, Alexandra P. Newman, Michelle Chang, Lauren E. Finn, Cameron Stainken, Anil T. Mangla, Patrick Eteme, Morgan Wieck, Alison Green, Alexandra Edmundson, Diana Reichbind, Vernell Brown, Laura Quiñones, Allison Longenberger, Elke Hess, Megan Gumke, Alicia Manion, Hannah Thomas, Carla A. Barrios, Adrianna Koczwara, Thelonious W. Williams, Marcia Pearlowitz, Moussokoura Assoumou, Alessandra F. Senisse Pajares, Hope Dishman, Cody Schardin, Xiong Wang, Kendalyn Stephens, Nakema S. Moss, Gurpalik Singh, Christine Feaster, Lindsey Martin Webb, Anna Krueger, Kristen Dickerson, Courtney Dewart, Bree Barbeau, Amelia Salmanson, Lawrence C. Madoff, Julie M. Villanueva, Catherine M. Brown, A. Scott Laney

**Affiliations:** Centers for Disease Control and Prevention, Atlanta, Georgia, USA (R. Gharpure, S. Sami, N. Hall, A. Foreman, R.T. Sabo, V.R. Brown, B. Brumfield, J.N. Ricaldi, C. Dewart, J.M. Villanueva, A.S. Laney);; Massachusetts Department of Public Health, Boston, Massachusetts, USA (J. Vostok, H. Johnson, P.L. Schubert, H. Shephard, L.C. Madoff, C.M. Brown);; Council of State and Territorial Epidemiologists Applied Epidemiology Fellowship, Atlanta (H. Shephard, A. Edmundson);; Georgia Institute of Technology, Atlanta (A.B. Conley);; New York City Department of Health and Mental Hygiene, New York, New York, USA (L. Zielinski, L. Malec);; New York State Department of Health, Albany, New York, USA (A.P. Newman);; Los Angeles County Department of Public Health, Los Angeles, California, USA (M. Chang, L.E. Finn);; California Department of Public Health, Richmond, California, USA (C. Stainken);; DC Health, Washington, DC, USA (A.T. Mangla, P. Eteme);; Rhode Island Department of Health, Providence, Rhode Island, USA (M. Wieck, A. Green);; Connecticut Department of Public Health, Hartford, Connecticut, USA (A. Edmundson, D. Reichbind);; CDC Foundation, Atlanta (D. Reichbind, C.A. Barrios, T.W. Williams);; Philadelphia Department of Public Health, Philadelphia, Pennsylvania, USA (V. Brown Jr., L. Quiñones);; Pennsylvania Department of Health, Harrisburg, Pennsylvania, USA (A. Longenberger, E. Hess);; Florida Department of Health, Tallahassee, Florida, USA (M. Gumke);; New Hampshire Department of Health and Human Services, Concord, New Hampshire, USA (A. Manion, H. Thomas);; Chicago Department of Public Health, Chicago, Illinois, USA (C.A. Barrios, A. Koczwara);; Maryland Department of Health, Baltimore, Maryland, USA (T.W. Williams, M. Pearlowitz);; Alexandria Health Department, Alexandria, Virginia, USA (M. Assoumou);; Virginia Department of Health, Richmond, Virginia, USA (A.F. Senisse Pajares);; Georgia Department of Public Health, Atlanta (H. Dishman);; Minnesota Department of Health, St. Paul, Minnesota, USA (C. Schardin, X. Wang);; North Carolina Department of Health and Human Services, Raleigh, North Carolina, USA (K. Stephens);; Alabama Department of Public Health, Montgomery, Alabama, USA (N.S. Moss);; Indiana Department of Health, Indianapolis, Indiana, USA (G. Singh, C. Feaster);; Colorado Department of Public Health and Environment, Denver, Colorado, USA (L.M. Webb);; Maine Center for Disease Control and Prevention, Augusta, Maine, USA (A. Krueger);; Ohio Department of Health, Columbus, Ohio, USA (K. Dickerson, C. Dewart);; Utah Department of Health, Salt Lake City, Utah, USA (B. Barbeau, A. Salmanson)

**Keywords:** COVID-19, severe acute respiratory syndrome coronavirus 2, SARS-CoV-2, coronaviruses, viruses, coronavirus disease, respiratory infections, disease outbreaks, multistate outbreak, zoonoses, breakthrough infections, COVID-19 vaccines, Massachusetts, United States

## Abstract

During July 2021, severe acute respiratory syndrome coronavirus 2 (SARS-CoV-2) B.1.617.2 variant infections, including vaccine breakthrough infections, occurred after large public gatherings in Provincetown, Massachusetts, USA, prompting a multistate investigation. Public health departments identified primary and secondary cases by using coronavirus disease surveillance data, case investigations, and contact tracing. A primary case was defined as SARS-CoV-2 detected <14 days after travel to or residence in Provincetown during July 3–17. A secondary case was defined as SARS-CoV-2 detected <14 days after close contact with a person who had a primary case but without travel to or residence in Provincetown during July 3–August 10. We identified 1,098 primary cases and 30 secondary cases associated with 26 primary cases among fully and non–fully vaccinated persons. Large gatherings can have widespread effects on SARS-CoV-2 transmission, and fully vaccinated persons should take precautions, such as masking, to prevent SARS-CoV-2 transmission, particularly during substantial or high transmission.

In recent months, the B.1.617.2 (Delta) variant of severe acute respiratory syndrome coronavirus 2 (SARS-CoV-2) has spread globally and has become the predominant circulating variant within the United States (*1*). Although the coronavirus disease (COVID-19) vaccines approved or authorized in the United States are highly effective (*2**–**4*), including against the Delta variant (*5**–**7*), several studies have indicated that variants of concern might be overrepresented among COVID-19 vaccine breakthrough infections (*8**,**9*) and that reverse transcription PCR cycle threshold values, which provide a crude correlation to the amount of virus in a sample, can be similar for vaccinated and unvaccinated persons infected with the Delta variant (*10**,**11*), although viral load might decrease more rapidly among vaccinated persons (*12*). Studies before Delta variant predominance suggested that the risk for onward transmission from vaccinated persons to household members might be decreased compared with transmission from unvaccinated persons (*13**,**14*). However, a more recent study during Delta predominance showed similar rates of household transmission from vaccinated and unvaccinated persons (*12*). In addition, previous studies of persons with COVID-19 vaccine breakthrough infections have indicated that illness might be more commonly asymptomatic or present with fewer symptoms than infections among non‒fully vaccinated persons (*15**,**16*).

In July 2021, after multiple, large public gatherings in Provincetown, Massachusetts, USA, a large outbreak of SARS-CoV-2 infections caused by the Delta variant was reported (*10*). Initial investigation by the Massachusetts Department of Public Health (MA DPH) identified 469 cases among Massachusetts residents during July 6–25; of these cases, 346 (74%) were characterized as COVID-19 vaccine breakthrough infections. We describe epidemiologic characteristics of the full multistate outbreak, document examples of secondary transmission, and assess whether illness differed by vaccination status.

## Methods

### Initial Outbreak and Public Health Response

The town of Provincetown, at the northern tip of Cape Cod in Massachusetts, has a population of ≈3,000 permanent residents and, during peak summer months, can reportedly reach a population size of up to 60,000 persons. During July 3–17, thousands of visitors from across the United States traveled to Provincetown and participated in large, densely packed indoor and outdoor gatherings marketed to adult male participants. Multiple continuous events were held at venues such as restaurants, bars, and guest houses. Local advisories at the time did not recommend mask wearing for fully vaccinated persons, and venues did not require participants to wear masks indoors.

By July 10, MA DPH received multiple reports of an increasing cluster of COVID-19 cases among Massachusetts residents who resided in or recently visited Provincetown, including cases among fully vaccinated persons. On July 14, Massachusetts state and local health officials responded to the increase in cases by expanding access to SARS-CoV-2 mobile testing and recommending testing for all persons who traveled to Provincetown since July 1 or had close contact with persons who showed positive test results for SARS-CoV-2, regardless of vaccination status. On July 15 and July 21, MA DPH issued Epidemic Information Exchange notifications to identify additional cases among residents of US public health jurisdictions outside Massachusetts.

### Case Definitions

For this investigation, a primary cluster-associated case was defined as detection of SARS-CoV-2 RNA or antigen in a respiratory specimen collected from a person <14 days after travel to or residence in Provincetown during July 3–17. A secondary case was detection of SARS-CoV-2 RNA or antigen in a respiratory specimen from a person without history of travel to or residence in Provincetown during July 3–August 10 that was collected <14 days after close contact (within 6 feet for a cumulative total of >15 minutes within a 24-hour period) with a person who had a primary case during their infectious period. The infectious period of a person who had a primary case was defined as 2 days before through 10 days after symptom onset or, if asymptomatic, 2 days before through 10 days after a positive test result. Persons were considered symptomatic if they reported any COVID-19-like symptom within 14 days before or after specimen collection (*17*).

Fully vaccinated persons were those who were >14 days after completion of all recommended doses of a US Food and Drug Administration authorized COVID-19 vaccine (2 doses of Pfizer/BioNTech [https://www.pfizer.com] or Moderna [https://www.modernatx.com], or 1 dose of Johnson & Johnson [https://www.jandj.com]) and who had documentation in their state immunization information system or self-report of vaccination details (including vaccine product and dates of receipt) during case investigation. Non–fully vaccinated persons were those who were partially vaccinated or unvaccinated or whose vaccination status was unknown. Partially vaccinated persons were those who had received only 1 dose of a 2-dose vaccine series or were <14 days after vaccine completion at the time of specimen collection; unvaccinated persons and persons with unknown status were those without documentation or self-attestation of vaccination. A COVID-19 vaccine breakthrough case was a cluster-associated case in a person who was fully vaccinated before collection of a SARS-CoV-2 positive specimen.

### Data Collection and Analysis

For this investigation, state and local public health departments identified primary cases by using travel history documented in their COVID-19 surveillance systems (capturing demographic data, previous COVID-19 illness, underlying medical conditions, vaccination history, symptoms, and clinical outcomes), as well as supplemental case investigation and contact tracing of persons who self-reported an association with the outbreak. Secondary cases were identified, to the extent feasible, through case investigation and contact tracing of primary cases. Self-reported underlying medical conditions associated with increased risk for severe COVID-19 included in this investigation were active cancer undergoing current treatment, autoimmune disease, cardiovascular disease, chronic kidney disease, chronic liver disease, chronic lung disease, current pregnancy, diabetes, solid organ or stem cell transplant, infection with HIV, and other immunocompromising conditions (*18*).

Case data collected by state and local health departments were sent to MA DPH; personally identifiable information was removed before sharing with CDC. We performed data collation and analysis by using SAS software version 9.4 (https://www.sas.com). This activity was reviewed by CDC and was conducted consistent with applicable federal law and CDC policy.

### Laboratory Testing

State/local public health laboratories and laboratory partners confirmed cases by using SARS-CoV-2 nucleic acid amplification test or antigen test. Laboratories used a variety of platforms to conduct testing and sequencing of available cluster-associated specimens; variant identification results were shared with MA DPH and subsequently with CDC. Sequences were uploaded to the GISAID database (*19*) or GenBank (*20*).

## Results

### Description of the Outbreak

**Figure 1:**
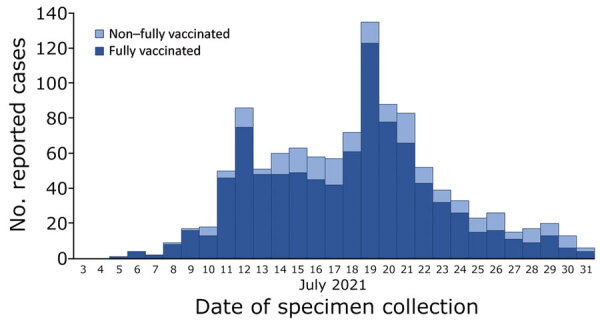
Primary cluster-associated cases of severe acute respiratory syndrome coronavirus 2 infection (n = 1,098), by vaccination status and date of specimen collection, after large public gatherings in Provincetown, Massachusetts, USA, July 2021. Fully vaccinated persons were those who were >14 days after completion of all recommended doses of a US Food and Drug Administration‒authorized coronavirus disease vaccine (2 doses of Pfizer/BioNTech [https://www.pfizer.com] or Moderna [https://www.modernatx.com], or 1 dose of Johnson & Johnson [https://www.jandj.com]), with documentation in their state immunization information system or self-report of vaccination details during case investigation. Non–fully vaccinated includes persons who were partially vaccinated or unvaccinated or whose vaccination status was unknown.

During July 5–31, 2021, a total of 1,098 persons who traveled to or resided in Provincetown during July 3–17 showed positive test results for SARS-CoV-2 (Figure 1). Of these, 625 (57%) were Massachusetts residents and 473 (43%) were visitors from 20 US states, predominantly New York (123, 26%) and California (87, 18%), as well as the District of Columbia (52, 11%) (Appendix Figure 1). Most primary cluster-associated cases were in men (88%), adults 19–49 years of age (66%), and non-Hispanic White persons (66%). Genomic sequencing of primary case specimens identified the B.1.617.2 (Delta) variant of SARS-CoV-2 in 364 (98%) of 371 sequenced specimens, the AY.3 sublineage (Delta) in 1 (0.3%), the AY.4 sublineage (Delta) in 3 (0.8%), and P.1 (Gamma) in 3 (0.8%).

### Secondary Transmission

**Figure 2:**
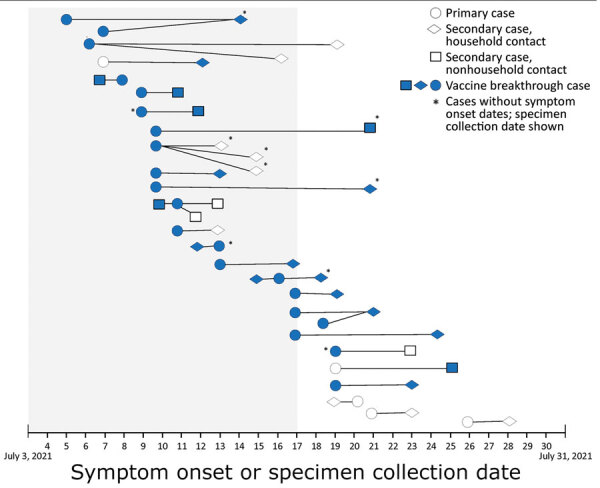
Transmission diagram of primary (n = 26) and secondary (n = 30) cluster-associated cases of severe acute respiratory syndrome coronavirus 2 (SARS-CoV-2) infection, by vaccination status and date of symptom onset or specimen collection, after large public gatherings in Provincetown, Massachusetts, USA, July 2021. A primary case was defined as detection of SARS-CoV-2 RNA or antigen in a respiratory specimen collected from a person <14 days after travel to or residence in Provincetown during July 3–17. A secondary case was defined as detection of SARS-CoV-2 RNA or antigen in a respiratory specimen collected from a person <14 days after close contact (within 6 feet for a cumulative total of >15 minutes within a 24-hour period) with a person who had a primary case during their infectious period, and without history of travel to or residence in Provincetown during July 3–August 10. The infectious period of a person with a primary case was defined as 2 days before through 10 days after symptom onset or, if asymptomatic, 2 days before or through 10 days after a positive test result. A vaccine breakthrough case was a cluster-associated case in a person who completed all recommended doses of a US Food and Drug Administration‒authorized COVID-19 vaccine (2 doses of Pfizer/BioNTech [https://www.pfizer.com] or Moderna [https://www.modernatx.com], or 1 dose of Johnson & Johnson [https://www.jandj.com]) >14 days before collection of a SARS-CoV-2‒positive specimen. Gray shading indicates the event exposure period (July 3–17, 2021) in the primary case definition. Only primary cases associated with a secondary case are shown. Symptom onset of persons with secondary cases before symptom onset of persons with primary cases was observed in 4 pairs, consistent with previous reports (*21*,*22*), and could be caused by presymptomatic transmission (*23*,*24*) or variability in self-reported symptom onset date. Household contacts were exposed to persons who had a primary case within household settings. Settings of nonhousehold exposures were workplace (1), summer camp (2), social gatherings (4), shared ride (1), and unknown (1).

We identified 30 secondary cases of SARS-CoV-2 infection in residents from 7 states; date of SARS-CoV-2–positive specimen collection ranged from July 11 through July 29, 2021, resulting in 1,128 cluster-associated cases (Table 1). Persons with secondary cases were epidemiologically linked to 26 persons who had primary cases (Figure 2). Eighteen (60%) of 30 secondary cases occurred in fully vaccinated persons, as did 21 (81%) of 26 primary cases; there were 16 primary/secondary case pairs in which both persons were fully vaccinated. Most persons who had secondary cases (21, 70%) were household contacts of persons who had primary cases.

**Table 1:** Demographic and clinical characteristics of persons who had primary and secondary cluster-associated cases of SARS-CoV-2 infection, by vaccination status, after large public gatherings in Provincetown, Massachusetts, USA, July 2021*

Characteristic	Overall, n = 1,128	Vaccination status†	
		Fully vaccinated, n = 918	Non–fully vaccinated, n = 210
Sex‡	n = 1,122	n = 914	n = 208
M	981 (87)	822 (90)	159 (76)
** F**	139 (12)	90 (10)	49 (24)
Age group, y	n = 1,096	n = 894	n = 202
<1–11	17 (2)	0	17 (8)
12–18	8 (0.7)	2 (0.2)	6 (3)
19–49	720 (66)	589 (66)	131 (65)
50–64	306 (28)	264 (30)	42 (21)
65–74	41 (4)	35 (4)	6 (3)
>75	4 (0.4)	4 (0.4)	0
Race/ethnic group	n = 1,058	n = 863	n = 195
Hispanic or Latino	70 (7)	42 (5)	28 (14)
Non-Hispanic White	694 (66)	615 (71)	79 (41)
Non-Hispanic Black	18 (2)	11 (1)	7 (4)
Non-Hispanic other race or multiracial	276 (26)	195 (23)	81 (42)
Residence	n = 1,128	n = 918	n = 210
Provincetown	253 (22)	171 (19)	82 (39)
Other area in Massachusetts	389 (34)	312 (34)	77 (37)
Outside Massachusetts	486 (43)	435 (47)	51 (24)
Previous COVID-19 diagnosis§	n = 383	n = 345	n = 38
Previous COVID-19 diagnosis	12 (3)	10 (3)	2 (5)
Duration since previous positive test result, d	n = 10	n = 8	n = 2
Median	205	205	300
Range	122–488	136–488	122–478
Interquartile range	177–434	186–367	211–389
Underlying medical conditions¶	n = 1,128	n = 918	n = 210
Any	130 (12)	118 (13)	12 (6)
Symptoms	n = 1,036	n = 873	n = 163
Asymptomatic	40 (4)	32 (4)	8 (5)
Symptomatic	996 (96)	841 (96)	155 (95)
Symptoms reported	n = 947	n = 799	n = 148
Abdominal pain	61 (6)	48 (6)	13 (9)
Chills	323 (34)	280 (35)	43 (29)
Congestion	524 (55)	463 (58)	61 (41)
Cough	667 (70)	582 (73)	85 (57)
Diarrhea	176 (19)	160 (20)	16 (11)
Difficulty breathing/shortness of breath	94 (10)	83 (10)	11 (7)
Fatigue	374 (39)	331 (41)	43 (29)
Fever	417 (44)	343 (43)	74 (50)
Headache	453 (48)	378 (47)	75 (51)
Loss of appetite	150 (16)	128 (16)	22 (15)
Loss of smell or taste	456 (48)	402 (50)	54 (36)
Muscle aches/pains	374 (40)	313 (39)	61 (41)
Sore throat	380 (40)	338 (42)	42 (28)
Vomiting	29 (3)	26 (3)	3 (2)
Symptom count	n = 947	n = 799	n = 148
Median	4	5	4
Range	1–13	1–13	1–12
Interquartile range	3–7	3–7	2–6
Time from symptom onset to specimen collection, d	n = 930	n = 783	n = 147
Median	2	2	2
Range	−5 to 16	−3 to 14	−5 to 16
Interquartile range	1–4	1–4	1–4
Clinical course	n = 1,128	n = 918	n = 210
Admitted to hospital	8 (0.7)	7 (0.8)	1 (0.5)
Duration of hospitalization, d			
Median	5	5	6
Range	2–39	2–39	NA
Interquartile range	3.75–6.25	3.5–6.0	NA
Admitted to intensive care	2 (0.1)	2 (0.2)	0

*Values are no. (%) unless indicated otherwise. Percentages might not total 100% because of rounding. Denominators for individual variables exclude cases that have missing data. COVID-19, coronavirus disease; NA, not applicable; SARS-CoV-2, severe acute respiratory syndrome coronavirus 2.

†Fully vaccinated persons were those who were >14 d after completion of all recommended doses of a US Food and Drug Administration‒authorized COVID-19 vaccine (2 doses of Pfizer/BioNTech [https://www.pfizer.com] or Moderna [https://www.modernatx.com], or 1 dose of Johnson & Johnson [https://www.jandj.com]), with documentation in their state immunization information system or self-report of vaccination details during case investigation. Non–fully vaccinated includes 39 persons who were partially vaccinated and 171 who were unvaccinated or whose vaccination status was unknown.

‡Gender responses of “Transgender/non-binary” (2; <1%) are not shown because of small counts.

§Previous COVID-19 was defined as detection of SARS-CoV-2 RNA or antigen in a respiratory specimen >90 d before collection of the cluster-associated specimen.

¶Persons who had underlying medical conditions associated with increased risk for severe COVID-19, including active cancer, autoimmune disease, cardiovascular disease, chronic kidney disease, chronic liver disease, chronic lung disease, current pregnancy, diabetes, solid organ or stem cell transplant, infection with HIV, and other immunocompromising conditions. Specific underlying conditions are described in Appendix Table 2.

The age distribution of persons with primary and secondary cases differed (Table 2); 5 children <12 years of age and 1 person >75 years of age had secondary cases. Equal proportions of persons with primary and secondary cases had symptomatic illness (96%). For 20 primary/secondary case pairs with reported symptom onset, the median time from primary to secondary symptom onset (serial interval) was 2 (range −1 to 13) days. Phylogenetic analyses of 3 available primary/secondary case pairs indicated that each case pair was genetically similar (Appendix Figure 2).

**Table 2:** Demographic and clinical characteristics of persons who had cluster-associated cases of SARS-CoV-2 infection, by primary and secondary case classification, after large public gatherings in Provincetown, Massachusetts, USA, July 2021*

Characteristic	Case classification†	
	Primary case, n = 26	Secondary case, n = 30
Sex	n = 26	n = 29
M	22 (85)	15 (52)
F	4 (15)	14 (48)
Age group, y	n = 26	n = 30
<1–11	0	5 (17)
12–18	0	0
19–49	20 (77)	16 (53)
50–64	5 (19)	8 (27)
65–74	1 (4)	0
≥75	0	1 (3)
Race/ethnic group	n = 24	n = 25
Hispanic or Latino	1 (4)	3 (12)
Non-Hispanic White	16 (67)	13 (52)
Non-Hispanic Black	1 (4)	0
Non-Hispanic other race or multiracial	6 (25)	9 (36)
Residence	n = 26	n = 30
Provincetown	0	0
Other area in Massachusetts	17 (65)	17 (57)
Not in Massachusetts	9 (35)	13 (43)
Vaccination status‡	n = 26	n = 30
Fully vaccinated	21 (81)	18 (60)
Previous COVID-19 illness§	n = 24	n = 29
Previous COVID-19 diagnosis	1 (4)	1 (3)
Duration since previous positive test result, d	193	196
Underlying medical conditions¶	n = 26	n = 30
Any	7 (27)	3 (10)
Symptoms	n = 25	n = 26
Asymptomatic	1 (4)	1 (4)
Symptomatic	24 (96)	25 (96)
Symptoms reported	n = 24	n = 22
Abdominal pain	3 (13)	1 (5)
Chills	13 (54)	7 (32)
Congestion	14 (58)	13 (59)
Cough	16 (67)	14 (64)
Diarrhea	6 (25)	5 (23)
Difficulty breathing/shortness of breath	3 (13)	1 (5)
Fatigue	14 (58)	13 (59)
Fever	17 (71)	10 (45)
Headache	11 (46)	13 (59)
Loss of appetite	10 (42)	4 (18)
Loss of smell or taste	12 (50)	13 (59)
Muscle aches/pains	14 (58)	8 (36)
Sore throat	10 (42)	9 (41)
Vomiting	1 (4)	0
Symptom count		
Median	6	5
Range	1–13	1–10
Interquartile range	4‒7	3–6
Time from symptom onset to specimen collection date, d	n = 23	n = 23
Median	2	2
Range	0–9	−1 to 6
Interquartile range	1–3	1–4
Clinical course	n = 26	n = 30
Admitted to hospital	1 (4)	0
Admitted to intensive care unit	0	0

*Values are no. (%) unless indicated otherwise. Percentages might not total 100% because of rounding. Denominators for individual variables exclude cases that have missing data. COVID-19, coronavirus disease; SARS-CoV-2, severe acute respiratory syndrome coronavirus 2.

†A primary case was defined as detection of SARS-CoV-2 RNA or antigen in a respiratory specimen collected from a person <14 d after travel to or residence in Provincetown during July 3–17. A secondary case was defined as detection of SARS-CoV-2 RNA or antigen in a respiratory specimen collected from a person <14 d after close contact (within 6 feet for a cumulative total of >15 min within a 24-h period) with a person who had a primary case during their infectious period, and without history of travel to or residence in Provincetown during July 3–August 10. Secondary cases do not include chains of transmission occurring within visitors/residents in Provincetown. The infectious period of a person with a primary case was defined as 2 d previously through 10 d after symptom onset or, if asymptomatic, 2 d previously through 10 d after a positive test result. Only primary cases associated with a secondary case are presented.

‡Fully vaccinated persons were those who were >14 d after completion of all recommended doses of a US Food and Drug Administration‒authorized COVID-19 vaccine (2 doses of Pfizer/BioNTech [https://www.pfizer.com] or Moderna [https://www.modernatx.com], or 1 dose of Johnson & Johnson [https://www.jandj.com]), with documentation in their state immunization information system or self-report of vaccination details during case investigation.

§Previous COVID-19 illness was defined as detection of SARS-CoV-2 RNA or antigen in a respiratory specimen >90 d before collection of the cluster-associated specimen.

¶Persons who had underlying medical conditions associated with increased risk for severe COVID-19, including active cancer, autoimmune disease, cardiovascular disease, chronic kidney disease, chronic liver disease, chronic lung disease, current pregnancy, diabetes, solid organ or stem cell transplant, infection with HIV, and other immunocompromising conditions (*13*).

### Characteristics of Vaccine Breakthrough and Non-Breakthrough Cases

Among the 1,128 cluster-associated primary and secondary cases, we identified 918 (81%) vaccine breakthrough cases. We confirmed vaccination status by matching to the state immunization information system for 664 (72%) cases and by self-report for 254 (28%) cases. Among fully vaccinated persons, most were men (90%), 19–49 years of age (66%), and non-Hispanic White (71%); a total of 13% had >1 underlying medical condition associated with increased risk for severe COVID-19 (Table 1). Among non‒fully vaccinated persons, 39 (19%) persons were partially vaccinated and 171 (81%) were unvaccinated or had unknown vaccination status.

**Figure 3:**
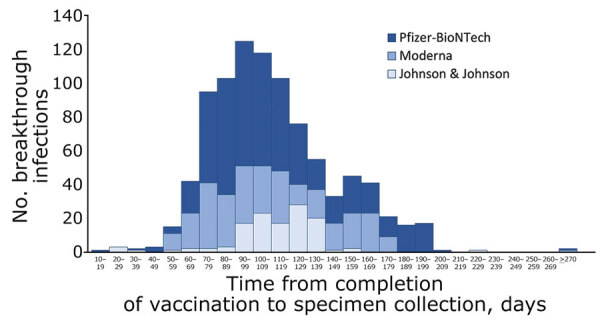
Time from completion of coronavirus disease (COVID-19) vaccination to date of specimen collection, by vaccine product, among fully vaccinated persons (n = 918) who had primary and secondary cluster-associated cases of severe acute respiratory syndrome coronavirus 2 infection after large public gatherings in Provincetown, Massachusetts, USA, July 2021. Fully vaccinated persons were those who were >14 days after completion of all recommended doses of a US Food and Drug Administration‒authorized COVID-19 vaccine (2 doses of Pfizer/BioNTech [https://www.pfizer.com] or Moderna [https://www.modernatx.com], or 1 dose of Johnson & Johnson [https://www.jandj.com]), with documentation in their state immunization information system or self-report of vaccination details during case investigation. Minimum time from completion of vaccination to specimen collection for persons who had breakthrough infections was 14 days. Median time from completion of vaccination to SARS-CoV-2‒positive specimen collection was 105 (range 15–326) days. Median times from completion to infection, by vaccine product, were 104 (range 15–326) days for persons who received the Pfizer-BioNTech vaccine, 104 (range 50–280) days for persons who received the Moderna vaccine, and 115 (range 23–225) days for persons who received the Johnson & Johnson/Janssen vaccine. Two persons were >270 days after vaccination at the time of specimen collection; 1 was vaccinated with Moderna 280 days before; and the other person with Pfizer-BioNTech 326 days before. Both persons were vaccinated through COVID-19 vaccine clinical trials.

Of the 918 persons who had breakthrough infections, 504 (55%) received the Pfizer/BioNTech vaccine, 293 (32%) received the Moderna vaccine, and 121 (13%) received the Johnson & Johnson vaccine. Characteristics of vaccine breakthrough cases were similar across vaccine products (Appendix Table 1). The median time from completion of vaccination to SARS-CoV-2–positive specimen collection was 105 (range 15–326) days (Figure 3). For all cases, 12 (3%) of 383 persons who had available data had a previous COVID-19 diagnosis: 10/345 (3%) fully vaccinated and 2/38 (5%) non–fully vaccinated persons (Table 1).

### Symptoms and Clinical Outcomes

For the 1,036 persons who had a cluster-associated case and who reported symptom data, 996 (96%) had a symptomatic illness with onset dates ranging from July 1 to July 31 (Table 1). A similar percentage of fully vaccinated (96%) and non–fully vaccinated (95%) persons reported symptomatic illness; cough was the most commonly reported symptom among both groups (72% in fully vaccinated, 57% in non–fully vaccinated). The number of symptoms reported was also similar across both groups; fully vaccinated persons reported a median of 5 symptoms during illness (range 1–13) and non–fully vaccinated persons reported 4 (range 1–12).

Eight persons were hospitalized and subsequently discharged, including 7 (0.7%) fully vaccinated persons (of whom 2 were admitted to the intensive care unit during hospitalization) and 1 (0.5%) non–fully vaccinated person. Of the hospitalized patients, 6 (75%) reported an underlying medical condition: 5 (71%) of 7 fully vaccinated persons and 1 (100%) of 1 non–fully vaccinated person. No deaths were reported.

## Discussion

This investigation highlights that the Delta variant of SARS-CoV-2 can spread quickly through a highly vaccinated population and can be transmitted to others regardless of vaccination status. Although vaccination remains a key mitigation strategy to decrease illness and death associated with COVID-19 (*25*), the Delta variant of SARS-CoV-2 is highly transmissible (*26*), and several studies have suggested lower vaccine effectiveness during Delta variant predominance compared with earlier months (*5**–**7**,**27*), probably driven by waning immunity from increased time since vaccination (*28*). In this outbreak, 99% of cluster-associated cases that had available sequencing were caused by the Delta variant, and 81% of cluster-associated cases were classified as vaccine breakthrough infections. The large number of breakthrough infections is probably representative of a highly vaccinated underlying population; as a greater proportion of the US population becomes fully vaccinated, vaccine breakthrough infections are likely to be more frequently observed (*27**,**29*).

Data from this outbreak provide support for an increasing body of evidence that fully vaccinated persons can transmit SARS-CoV-2 to others, including other fully vaccinated persons, particularly during Delta variant predominance (*12*,*15*,*30*). The observed examples of secondary transmission, particularly to children <12 years of age and to older persons >75 years of age, highlight that fully vaccinated persons should wear a mask indoors in public to reduce the risk for infection and prevent SARS-CoV-2 transmission, especially if they have someone in their household who is immunocompromised, at increased risk for severe disease, or not fully vaccinated (*31*). The serial interval between primary and secondary case onset (median 2 days) was comparable to what has been previously described for Delta variant transmission (median 2–3 days) (*21**,**22*). However, further characterization of serial interval, particularly stratified by vaccination status, is warranted. Symptom onset of persons who had secondary cases before symptom onset of persons who had primary cases was observed in a small number of pairs, consistent with previous reports (*21**,**22*), and could be caused by presymptomatic transmission (*23**,**24*) or variability in self-reported symptom onset date.

In this outbreak, most fully vaccinated and non–fully vaccinated persons were symptomatic, and the number of symptoms reported was similar between the 2 groups. This finding differs from those of previous studies that had limited data on Delta variant infections, which found that persons with vaccine breakthrough infections had fewer symptoms compared with persons who had non–breakthrough infections (*15*,*16*). In addition, hospitalizations were rare for fully vaccinated and non–fully vaccinated persons during this outbreak (<1%). Previous analyses have demonstrated that high effectiveness of COVID-19 vaccines against severe disease caused by the Delta variant, including hospitalization (*27*,*32*,*33*). Additional population-level surveillance of the clinical picture and outcomes of patients with Delta variant breakthrough infections is warranted to clarify differences in disease severity, including older adults and persons who have underlying conditions or other characteristics that might affect immune response to vaccination or predispose them to more severe COVID-19 illness. Additional studies are also needed to characterize the effect of vaccination on risk for reinfection with SARS-CoV-2. Previous studies have indicated that vaccination might reduce the risk for reinfection (*34*). However, the number of persons who had a previous COVID-19 diagnosis was inadequate to enable comparison in our study.

The first limitation of our study is that, because the outbreak occurred among an open population that included thousands of persons who traveled to Provincetown and whose infection and vaccination status were unknown, these data cannot be used to calculate or infer vaccine effectiveness or to compare COVID-19 vaccine products. Symptoms and outcomes observed in this investigation might be affected by greater presence of older age and underlying conditions for fully vaccinated persons compared with non–fully vaccinated persons.

Second, data abstracted from public health department surveillance systems can differ in method of collection and completeness of data. Although data were cleaned and combined across jurisdictions, bias might have been introduced if data were not missing at random (e.g., if persons who had unknown vaccination data more commonly had missing data for additional variables).

Third, vaccination status was assigned through matching with an immunization information system or self-report; persons who did not have vaccination data were assigned as non‒fully vaccinated, which could lead to misclassification bias. Symptom data, including date of onset, and underlying medical conditions were self-reported and might be incomplete or inaccurate.

Fourth, asymptomatic SARS-CoV-2 infections might be underrepresented; although testing recommendations in Massachusetts were changed on July 14 to encourage all persons, regardless of vaccination status, to seek testing after travel to Provincetown or close contact with a person who showed positive results for COVID-19, symptomatic persons might have been more likely to seek testing than asymptomatic persons because of previous CDC guidance that most asymptomatic vaccinated persons can refrain from testing. Consequently, the cluster was probably larger than documented, particularly underestimating asymptomatic infections. Similarly, attitudes, such as willingness to seek testing and report symptoms might have differed by vaccination status, potentially leading to greater case ascertainment and increased symptom prevalence among persons who had vaccine breakthrough cases.

Finally, the number of secondary cases might be greatly underestimated because capacity and methods for contact tracing and case follow-up varied across jurisdictions, particularly during the nationwide surge in COVID-19 cases attributed to the Delta variant. The frequency or attack rate of secondary transmission of SARS-CoV-2 cannot be inferred from these data. In addition, our investigation could not account for additional sources of SARS-CoV-2 exposure that could have led to infection among persons who had secondary cases. Furthermore, for this investigation, secondary cases only included those in persons who did not travel to Provincetown; additional chains of transmission occurring within visitors/residents in Provincetown are not described in this study.

In conclusion, major epidemiologic questions about breakthrough infections, such as the comparative infectiousness of fully vaccinated and non–fully vaccinated persons, duration of viral shedding, and duration of vaccine-derived immunity, remain. However, our findings underscore the need for persons who are fully vaccinated to take precautions to prevent transmission of SARS-CoV-2 to themselves and others, such as wearing a mask in public indoor settings or crowded outdoor settings, particularly during substantial or high transmission. Vaccination, although critical to reduce illness and death from COVID-19, should be complemented by layered mitigation strategies to address the COVID-19 pandemic (*25*,*31*).

Appendix Additional information on multistate outbreak of SARS-CoV-2 infections, including COVID-19 vaccine breakthrough infections, associated with large public gatherings, United States.
