# Supplementary material for: Multistate Outbreak of SARS-CoV-2 Infections, Including Vaccine Breakthrough Infections, Associated with Large Public Gatherings, United States
Source: Emerg Infect Dis. 2022 Jan;28(1):35–43. doi: 10.3201/eid2801.212220 (PMC8714214; doi:10.3201/eid2801.212220)
Supplement: Appendix — Additional information on multistate outbreak of SARS-CoV-2 infections, including COVID-19 vaccine breakthrough infections, associated with large public gatherings, United States. [file 21-2220-Techapp-s1.pdf]

Article DOI: https://doi.org/10.3201/eid2801.212220

Multistate Outbreak of SARS-CoV-2 Infections, Including Vaccine Breakthrough Infections, Associated with Large Public Gatherings, United States

Appendix

Additional Methods

Phylogenetic Analysis of Primary/Secondary Case Pairs

There were 3 primary/secondary case pairs for which both specimens were sequenced; sequences from these pairs, as well as a non-outbreak Massachusetts sequence from a similar time period obtained from GISAID (*1*) and a reference sequence (Wuhan-Hu-1) from National Center for Biotechnology Information GenBank (*2*), were aligned by using multiple sequence comparison by log-expectation (MUSCLE) (*3*). The alignment was used to create a maximum-likelihood tree in MEGA X version 10.1.8 (https://www.megasoftware.net); 500 bootstrap replicates were performed. Pairwise distances were also obtained from MEGA X.

References

1. Elbe S, Buckland-Merrett G. Data, disease and diplomacy: GISAID's innovative contribution to global health. Glob Chall. 2017;1:33–46. PubMed https://doi.org/10.1002/gch2.1018

2. NCBI Resource Coordinators. Database resources of the National Center for Biotechnology Information. Nucleic Acids Res. 2016;44(D1):D7–19. PubMed https://doi.org/10.1093/nar/gkv1290

3. Madeira F, Park YM, Lee J, Buso N, Gur T, Madhusoodanan N, et al. The EMBL-EBI search and sequence analysis tools APIs in 2019. Nucleic Acids Res. 2019;47(W1):W636–41. PubMed https://doi.org/10.1093/nar/gkz268

4. Centers for Disease Control and Prevention. COVID-19: people with certain medical conditions, August 2021 [cited 2021 Nov 7]. https://www.cdc.gov/coronavirus/2019-ncov/need-extra-precautions/people-with-medical-conditions.htm

Appendix Table 1. Characteristics of fully vaccinated persons who had cluster-associated cases of SARS-CoV-2 infection (n = 918), by vaccine product*

Characteristic	Vaccine product, no. (%)					
	Pfizer, n = 504		Moderna, n = 293		Johnson and Johnson/Janssen, n = 121	
Sex†	502		292		120	
M	452	(90)	261	(89)	109	(91)
F	49	(10)	30	(10)	11	(9)
Age, y	487		288		119	
12–18	2	(0.4)	0	(0)	0	(0)
19–49	328	(67)	177	(61)	84	(71)
50–64	142	(29)	92	(32)	30	(25)
65–74	14	(3)	16	(6)	5	(4)
≥75	1	(0.2)	3	(1)	0	(0)
Race/ethnic group	473		278		114	
Hispanic/Latino	24	(5)	13	(5)	5	(4)
White, non-Hispanic	338	(71)	195	(70)	82	(72)
Black, non-Hispanic	4	(0.8)	4	(1)	3	(3)
Multiracial/other race, non-Hispanic	105	(23)	66	(24)	24	(21)
Previous COVID-19‡	220		81		44	
History of COVID-19 diagnosis	5	(2)	3	(4)	2	(5)
Duration of time since previous positive test result, d	3		2		2	
Median	191		312		264.5	
Range	172–214		136–488		196–333	
Interquartile range	182–203		302–478		230–299	
Underlying medical condition§	504		293		121	
Any	75	(15)	34	(12)	9	(7)
Symptoms	483		280		110	
Asymptomatic	10	(2)	17	(6)	5	(5)
Symptomatic	473	(98)	263	(94)	105	(96)
Symptoms reported	446		254		99	
Abdominal pain	31	(7)	14	(6)	3	(3)
Chills	172	(39)	67	(26)	41	(41)
Congestion	262	(59)	156	(61)	45	(45)
Cough	338	(76)	177	(70)	67	(68)
Diarrhea	87	(20)	57	(22)	16	(16)
Difficulty breathing/shortness of breath	48	(11)	20	(8)	15	(15)
Fatigue	190	(43)	101	(40)	40	(40)
Fever	193	(43)	92	(36)	58	(59)
Headache	209	(47)	124	(49)	45	(45)
Loss of appetite	70	(16)	36	(14)	22	(22)
Loss of smell or taste	232	(52)	122	(48)	48	(48)
Muscle aches/pains	183	(41)	86	(34)	44	(44)
Sore throat	208	(47)	91	(36)	39	(39)
Vomiting	15	(3)	7	(3)	4	(4)
Symptom count	446		254		99	
Median	5		4		5	
Range	1–13		1–12		1–10	
Interquartile range	3–7		2–6		3–7	
Time from symptom onset to specimen collection date, d	444		241		98	
Median	2		2		2	
Range	−3 to 14		−3 to 12		−1 to 14	
Interquartile range	1–4		1–4		1–4	
Clinical course	504		293		121	
Admitted to hospital	5	(1)	0	(0)	2	(2)
Duration of hospitalization, d	4		0		2	
Median	4		NA		5	
Range	3–5		NA		2–7	
Interquartile range	1		NA		3	
Admitted to intensive care unit	1	(0.2)	NA		1	(0.8)

*Values are no.(%) unless indicated otherwise. Percentages might not total 100% because of rounding. Denominators for individual variables exclude cases that had missing data. COVID-19, coronavirus disease; NA, not applicable; SARS-CoV-2, severe acute respiratory syndrome coronavirus 2.
†Sex responses of Transgender/non-binary (2; <1%) are not shown because of small counts.
‡Previous COVID-19 was defined as detection of SARS-CoV-2 RNA or antigen in a respiratory specimen ≥90 d before collection of the cluster-associated specimen.
§Persons who had underlying medical conditions associated with increased risk for severe Covid-19, including active cancer, autoimmune disease, cardiovascular disease, chronic kidney disease, chronic liver disease, chronic lung disease, current pregnancy, diabetes, solid organ or stem cell transplant, infection with HIV, and other immunocompromising conditions (*4*).

Appendix Table 2. Underlying medical conditions* for persons who have cluster-associated Cases of SARS-CoV-2 infection (n = 1,128), by vaccination status*

	Vaccination status‡					
	Overall, n = 1,128		Fully vaccinated, n = 918		Non–fully vaccinated, n = 210	
Underlying medical condition†	no./total no.	%	no./total no.	%	no./total no.	%
Any underlying medical condition	130/1,128	12	118/918	13	12/210	6
Active cancer	3/44	7	3/42	7	0/2	0
Autoimmune disease	11/85	13	11/79	14	0/6	0
Cardiovascular disease	38/111	34	36/101	37	2/10	20
Chronic kidney disease	3/85	4	3/77	4	0/8	0
Chronic liver disease	4/86	5	4/78	5	0/8	0
Chronic lung disease	25/99	25	22/89	25	3/10	30
Current pregnancy	4/49	8	4/47	9	0/2	0
Diabetes	25/101	2	21/89	24	4/12	33
Hematopoietic stem cell transplant	0/38	0	0/36	0	0/2	0)
HIV infection	6/63	1	6/59	10	0/4	0
Solid organ transplant	1/42	2	1/40	3	0/2	0
Other immunocompromising condition	46/125	37	41/112	37	5/13	38

*Denominators for individual variables exclude cases with missing data.
†Includes underlying medical conditions associated with increased risk for severe coronavirus disease (*4*).
‡Fully vaccinated persons were those who were ≥14 d after completion of all recommended doses of a US Food and Drug Administration–authorized coronavirus disease vaccine (2 doses of Pfizer-BioNTech or Moderna, or 1 dose of Janssen [Johnson and Johnson]), with documentation in their state immunization information system or self-report of vaccination details during case investigation. Non–fully vaccinated includes 39 persons who were partially vaccinated and 171 who were unvaccinated or whose vaccination status was unknown.

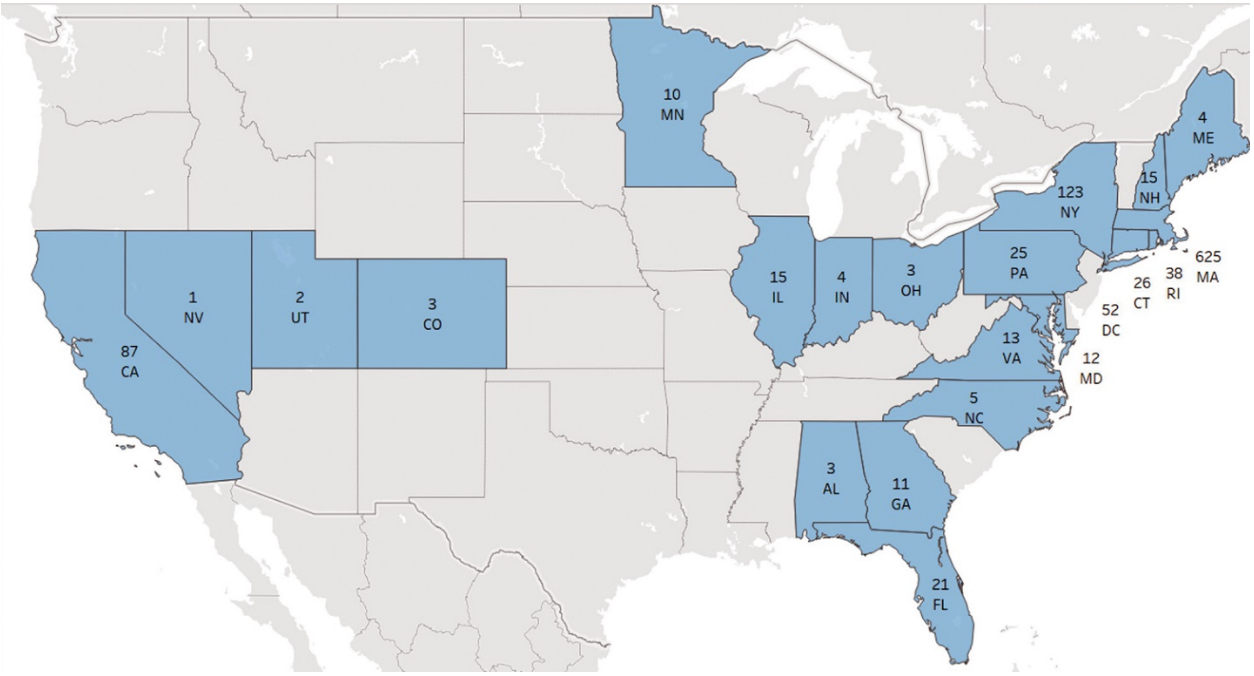

Appendix Figure 1. Jurisdiction of residence of persons who had primary cluster–associated cases of severe acute respiratory syndrome coronavirus 2 infections (n = 1098). AL, Alabama; CA, California; CO, Colorado; CT, Connecticut; DC, District of Columbia; FL, Florida; GA, Georgia; IL, Illinois; IN, Indiana; MA, Massachusetts; MD, Maryland; ME, Maine; MN, Minnesota; NC, North Carolina; NH, New Hampshire; NV, Nevada; NY, New York; OH, Ohio; PA, Pennsylvania; RI, Rhode Island; UT, Utah; VA, Virginia. Jurisdiction of residence includes 21 US states and DC.

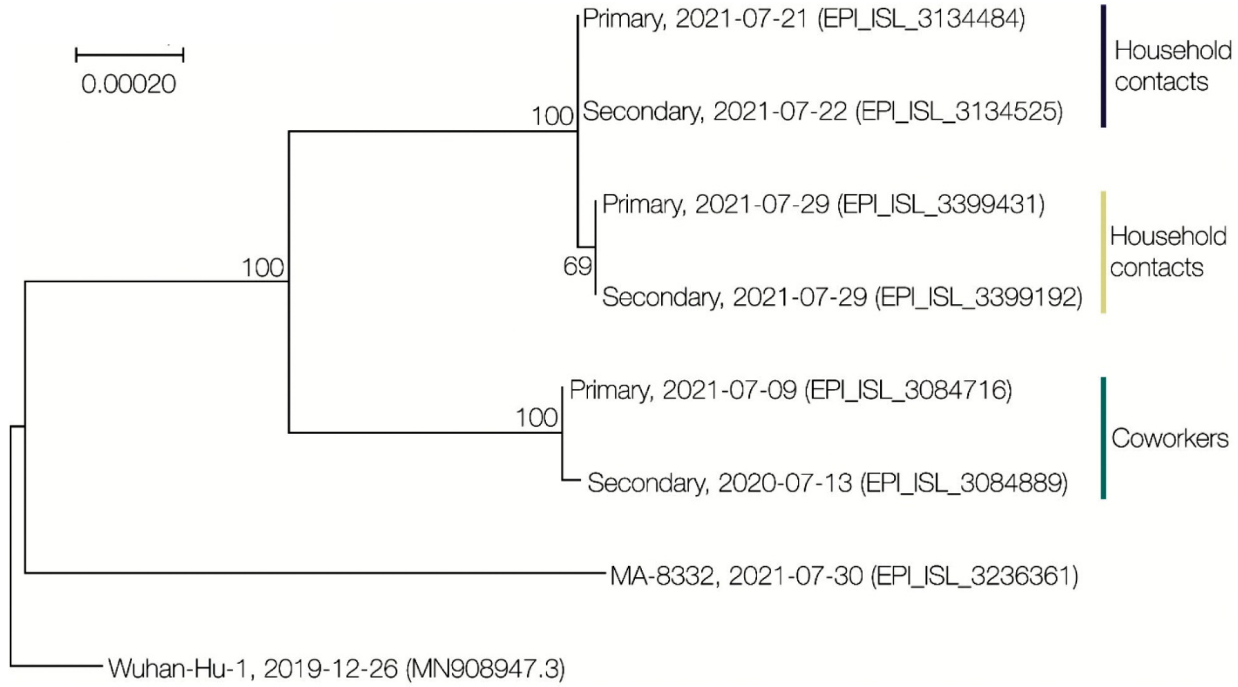

Appendix Figure 2. Phylogenetic analysis of transmission pairs (n = 3) cluster–associated cases of severe acute respiratory syndrome coronavirus 2 infections. Phylogenetic tree includes sequences for 3 transmission pairs (primary and secondary cluster-associated cases), including 2 pairs of household contacts and 1 pair of co-workers. Cluster-associated sequences and a non–outbreak-associated Massachusetts sequence (MA-8332) from a similar time period were obtained from GISAID; an additional reference sequence (Wuhan-Hu-1) was obtained from the National Center for Biotechnology Information GenBank. Bootstrap support percentages (500 replicates of the maximum-likelihood tree) are shown on the branches. Scale bar indicates nucleotide substitutions per site. All sequences are publicly available by using the accession numbers shown. All 3 pairs were genetically similar with 0–1 single-nucleotide polymorphisms between them.
